# Building a Model of Advocacy: Improving the Dementia Capability of Managed Care Health Plans in California

**DOI:** 10.1093/geroni/igab046.059

**Published:** 2021-12-17

**Authors:** Brooke Hollister, Jarmin Yeh, Leslie Ross, Jennifer Schlesinger, Debra Cherry

**Affiliations:** 1 University of California, San Francisco, California, United States; 2 University of California, San Francisco, San Francisco, California, United States; 3 Alzheimer’s Los Angeles, Los Angeles, California, United States

## Abstract

Given the growing prevalence of Alzheimer’s Disease and related dementias, and the intensity of this population’s care needs, it is imperative that health plans (HPs) increase their dementia-capability. The Dementia Cal MediConnect (Dementia CMC) project proposes an innovative model of health care advocacy that can create dementia-capable systems change. The Dementia CMC project was a partnership (2013 – 2018) between local Alzheimer’s organizations and ten managed care HPs in California. It used the following model of health care advocacy: 1) Identify dementia-capable best practices to set as systems change indicators; 2) Identify and leverage public policies in support of systems change indicators; 3) Identify and engage champions; 4) Develop and advocate for a value and business case to improve dementia care; 5) Identify gaps in dementia-capable practices; 6) Provide technical assistance, tools, and staff training to address the gaps in dementia-capable practices; and 7) Track systems change. Systems change data was collected through participant observation with HPs and interviews with key informants. HPs reported making systems changes toward more dementia-capable practices such as: better pathways for identification and diagnosis; better identification, assessment, support, and engagement of caregivers; and improved systems of referral to Alzheimer’s organizations. Some indicators of systems change were inconclusive as a result of variability in HP practices and the lack of common record systems between HPs and providers. The application of this advocacy model has led to systems changes that can be replicated to improve care for people living with dementia and their caregivers.

